# Measuring Quality of Life in Stroke Survivors: Validity and Reliability of the Spanish COOP/WONCA Scale

**DOI:** 10.3390/medicina61050878

**Published:** 2025-05-12

**Authors:** Trinidad Sentandreu-Mañó, Adrián García-Mollá, Inmaculada Oltra Ferrús, José M. Tomás, José Ricardo Salom Terrádez

**Affiliations:** 1 Department of Physiotherapy, University of Valencia, 46010 Valencia, Spain; trinidad.sentandreu@uv.es (T.S.-M.); inmaoltraferrus@gmail.com (I.O.F.); 2 Department of Methodology of Behavioral Sciences, University of Valencia, 46010 Valencia, Spain; adrian.garcia-molla@uv.es; 3 Salom & Cabezas Clinic—Remed Medical Center, 46007 Valencia, Spain; salom.joster@gmail.com

**Keywords:** health-related quality of life, assessment, stroke survivors, COOP/WONCA

## Abstract

*Background and Objectives*: Strokes significantly impact patients’ quality of life (QoL), affecting physical, cognitive, and social functioning. While health-related quality of life (HRQoL) assessments are essential for patient-centered care and clinical decision-making, many existing tools are limited by cultural biases or practical constraints. The COOP/WONCA charts offer a simple, brief, and validated alternative for functional assessment, yet their psychometric properties in stroke survivors remain underexplored. This study aims to evaluate the validity and reliability of the COOP/WONCA charts in assessing HRQoL among Spanish stroke survivors. *Materials and Methods*: Structural validity, reliability, and criterion-related validity were studied for COOP/WONCA charts in a sample of 91 stroke survivors, where 39.6% were women and 60.4% were men. Their ages ranged from 44 to 93 years old (M  =  68.84; SD = 9.44). A total of 70% of the strokes had an ischemic origin and 30% were hemorrhagic. To assess dimensionality, three Confirmatory Factor Analyses (CFA) were performed, differentiated by the inclusion of charts. *Results*: The CFA corroborate the best fitting of single-factor structure six-charts version (χ^2^ (9) = 8.69, *p* = 0.465, RMSEA = 0.000, 90% CI [0.000–0.141], CFI = 0.999, SRMR = 0.048). The results of criterion-related validity indicated significant correlation between dimensions of SF-36 and sociodemographic, clinical and physical variables. Cronbach’s alpha was 0.95 for the 9 domains version and 0.93 for the 6 domains version. *Conclusions*: These findings support that the COOP/WONCA Spanish version is a reliable and valid scale for assessing health-related quality of life among stroke survivors.

## 1. Introduction

In recent years, Spain and other developed countries have experienced a decline in stroke mortality rates, attributed to improvements in living conditions and advancements in healthcare systems. Despite these advances, approximately 13.7 million individuals worldwide experience a first stroke each year [1], and this number is projected to increase to 23 million globally by 2030, which is expected to result in a corresponding increase in the prevalence of stroke survivors [2]. Between 2016 and 2018, a total of 209,799 hospital admissions for ischemic stroke were recorded in Spain, corresponding to an average incidence rate of 150.17 cases per 100,000 population [3].

Stroke remains an important public health concern, requiring immediate medical and psychological attention [4]. There is a general recognition regarding the importance of including measures of quality of life (QoL) in stroke patients [4]. Physical, social, and cognitive impairment following a stroke can negatively affect life satisfaction and QoL [5,6]. Health-related quality of life (HRQoL) measures provide a comprehensive patient-centered approach and contain the individual patients’ reports on the impact of the stroke and its treatment on different domains of their daily life and health experience [7]. The perception of quality of life of stroke survivors can be a dynamic and changing process that depends on the coverage of subjectively demanded needs in a specific sociocultural context and throughout the different phases of evolution and recovery [8]. This transition is critical in a complex physical and psychological process of adaptation to a different condition [9]. Assessing HRQoL adds relevant information for treatment decision-making, efficacy evaluation, interpretation of outcomes and, in addition, it may help to estimate the needs, resource allocation and policy [7,10]. Several studies suggest that subjective evaluations of health may be better predictors of mortality than the severity of health problems diagnosed [11]. Despite this, a recent systematic review emphasized existing gaps in the evidence regarding the psychometric properties of patient-reported outcome measures of HRQoL in stroke populations [12]. These findings highlight the necessity for ongoing efforts to validate and implement standardized QoL assessment tools in stroke populations, ensuring both research and clinical practices are aligned with patient-centered outcomes.

On the other hand, little is known about the HRQoL of stroke patients, and the affected HRQoL domains appear to vary geographically and/or culturally [13]. Some examples of HRQoL instruments used in stroke survivors include generic measures such as the SF-36 or the European Quality of Life (EuroQol) and stroke-specific ones such as the Stroke Impact Scale (SIS), Newcastle Stroke-Specific Quality of Life Measure (NEWSQOL), and the Health-Related Quality of Life in Stroke Patients (HRQoLISP), among others. There are strengths and limitations to each approach. In the first group, the noncompletion bias and marked floor and ceiling effects may limit their utility. In the case of the instruments for stroke, they are criticized in some cases for a lack of cross-cultural validity, or for the complexity and long duration of their application, among other limitations [14,15,16]. Having a suitably comprehensive assessment that is sensitive to the nuances of QoL against the time and burden required for this assessment needs to be balanced [14]. Features such as time spent, simplicity, brevity and clarity of the questions, as well as the ease of correction, interpretation and presentation of the results, are some of the characteristics relevant for its implementation in clinical practice [17]. The COOP/WONCA charts are an instrument that could present these characteristics. From the beginning, these charts were developed in order to obtain an easy, feasible, valid and reliable instrument for use in daily clinical practice [11,17]. The World Organization of National Colleges, Academies and Academic Associations of General Practitioners (WONCA) selected this questionnaire as the most appropriate for measuring functional status at the international level, and this decision was based on the nature of the questionnaire as well as the published psychometric properties [18].

The COOP/WONCA charts have been adapted and validated for the Spanish population [14]. There are studies on psychometric properties of the questionnaire in different population groups, such as patients with osteoarthritis [19] and chronic renal pathology [20,21], with good results. In other countries, this instrument has also been validated in populations with chronic cardiorespiratory pathology [22,23], lumbar pathology [24], mental illness [25,26], migraine [27], oncological disease [28] and neurological problems [29,30,31,32,33]. To our knowledge, there is only one study that has studied psychometric characteristics of this scale (English version) in patients that survived a stroke, but the sample was made up only of patients with ischemic stroke of more than one year of evolution [32].

Therefore, taking into account the lack of literature regarding the usefulness of this instrument in this specific population, the aim of this study is to analyze the validity and reliability of the COOP/WONCA charts as a measurement instrument for HRQoL in a sample of Spanish stroke survivors.

## 2. Materials and Methods

### 2.1. Study Design and Participants

This cross-sectional survey included 91 stroke survivors. The target population consisted of post-stroke and community-dwelling adults who were recruited from recreational clubs, associations, and health units. Inclusion criteria included a clinical diagnosis of stroke and a Mini-Mental State Examination (MMSE) score of 18 points or higher, permitting the participant to follow basic instructions and be collaborative during completion of the survey. Individuals with aphasia were included, provided that they demonstrated sufficient comprehension to follow simple commands and complete the questionnaire with the visual support supplied by the images in the COOP/WONCA charts. The exclusion criteria were acute disease and unstable chronic disease. This study was approved by the Ethics Committee for Human Research at the University of Valencia (No. H1545056527043) and carried out in accordance with the provisions of the Declaration of Helsinki. All participants provided written informed consent.

Given the approximate size of the eligible population, with an average of 6000 new cases annually in the Valencian Community [34], and considering the most unfavorable scenario of *p* = q = 0.5, a 95% confidence level was used. Given the difficulty in obtaining very large samples of individuals affected by stroke, a sample size of around 100 participants was determined. This sample size was deemed appropriate to ensure the proper execution of the necessary statistical and psychometric analyses for the scale’s adequate validation. The sample heterogeneity was justified in order to ensure alignment with this study’s objectives and to capture the variability of patient characteristics, aimed at exploring the applicability of the instrument across different patient profiles.

### 2.2. Measurements and Data Collection

#### 2.2.1. Health-Related Quality of Life

In order to assess HRQoL, COOP/WONCA charts and the Spanish Short Form 36 (SF-36) questionnaire were used [35]. The Spanish version of the COOP/WONCA charts [18] has nine charts: physical condition, feelings, daily activities, social activities, change in health, overall health, pain, social support, and quality of life. Each of these charts has a title, a question referring to health status, and five response options. Each option is illustrated with a drawing that represents the functional level in a five-point Likert scale. Higher scores represent worse functioning. Each chart deals with one of the aforementioned dimensions of quality of life, and the score of each chart allows for a direct interpretation of the measured domain [36]. The SF-36 is one of the most employed generic instruments to assess quality of life related to health (HRQoL) [16]. This HRQoL tool consists of 36 questions subdivided into eight domains: physical functioning, physical role, bodily pain, general health, vitality, social functioning, emotional role, and mental health. Higher scores are associated with a better perception of health condition.

#### 2.2.2. Socio-Demographic and Clinical Data Form

This part of the survey had questions defining the socio-demographic characteristics and stroke-related variables of the sample. Information such as age, gender, Body Mass Index (kg/m^2^), dominant hand, and co-morbidity was included. Moreover, clinical characteristics of the sample and other variables related to stroke such as risk factors, time post-stroke, hemiparesis side, and ischemic or hemorrhagic stroke, among others, were also collected. Cognitive function was measured by MMSE [37].

#### 2.2.3. Motor Impairment

Distal upper limb range of motion was measured by means of a goniometer [38]. Handgrip and pinch strength (kg_f_) was assessed with a Jamar dynamometer (Sammons and Preston INC, Bolingbrook, IL, USA). Three repetitions were done, leaving a resting period of 1 min between them and taking the highest of the three values [39,40]. Hypertonia of the distal upper limb was measured using the Modified Ashworth Scale [41,42]. The assessment started 5 min after laying the subject down in the supine position and using a single passive movement to evaluate each muscle group [43].

#### 2.2.4. Functional Measurements

Manual dexterity was assessed with Box and Block Test following the standardized protocol [44] and functional independence in activities of daily living (ADL) by Barthel Index [45]. 

### 2.3. Statistical Analysis

Three Confirmatory Factor Analyses (CFA) were performed in order to assess the factorial structure of the COOP/WONCA versions: nine-charts, reduced dimensions and six-charts versions. The estimation method employed in this study was WLSMV, given that it performs well when using categorical non-normal data [46]. Therefore, the following indexes were included: the chi-square statistic (χ^2^), the Comparative Fit Index (CFI), the Standardized Root Mean Square Residual (SRMR), and the Root Mean Squared Error of Approximation (RMSEA). Acceptable model fit is considered in the presence of CFI values equal to or greater than 0.90 and RMSEA values equal to or lower than 0.08 [47]. Analyses were performed using Mplus version 8.11 ((Muthén & Muthén, Los Angeles, CA, USA) [48]. Descriptive statistics and correlation coefficients were calculated in SPSS version 26 (IBM, Armonk, NY, USA). Correlations were also calculated between COOP/WONCA and SF-36. Finally, internal consistencies of the COOP/WONCA versions were estimated with Cronbach’s alphas.

## 3. Results

### 3.1. Characteristics of the Participants

With regard to participants’ characteristics, the current study includes 91 participants affected by stroke, of which 39.6% were women and 60.4% were men. A total of 70% of the strokes had an ischemic origin and 30% were hemorrhagic. Regarding the time post-stroke, 2.2% of the sample were in acute phase, 35.2% subacute and 62.6% were chronic. Age ranged from 44 to 93 years. With respect to the anthropometric characteristics, the average Body Mass Index was 26.16 ± 3.22. Right hemiparesis occurred for 41.8%, while 58.2% presented left hemiparesis. Time post-stroke ranged between 1 month and 18 years. All of them presented a higher MMSE than 18 points, the average being 28.64 (SD = 4.15). A total of 9.9% had mild aphasia as a related pathology. A total of 57.1% of the sample referred to pain, mostly located in the shoulder (53.8%). In addition, 39.6% of the participants did not show shoulder motor control. Table 1 and Table 2 show the main characteristics of the sample.

### 3.2. Dimensionality

**Figure 1:**
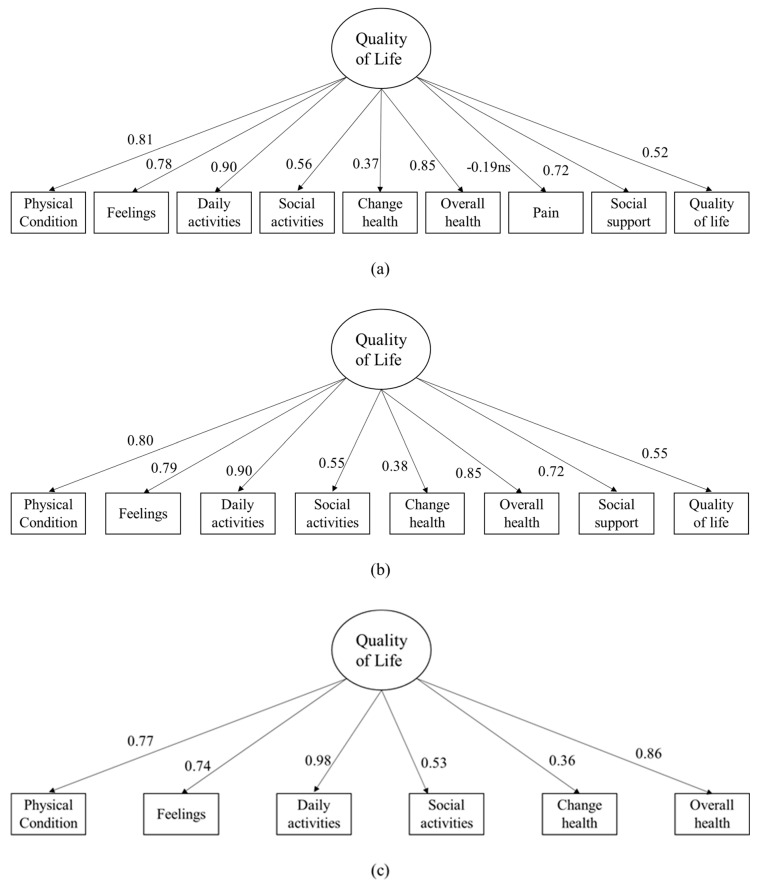
Standardized estimates for the Confirmatory Factor Analyses for the 9 charts of the COOP/WONCA (**a**), for a reduced factor of the COOP/WONCA (**b**), and finally, for the six charts of the COOP/WONCA (**c**). Note: All parameter estimates statistically significant (*p* < 0.01) unless ns stated (*p* > 0.05).

The dimensionality of the nine charts of the COOP/WONCA as indicators of an overall construct of quality of life has been studied by means of a CFA, in which a single latent variable of quality of life explains the nine indicators of quality of life. A graphical representation of this CFA is shown in Figure 1a. This model fitted the data reasonably well: χ^2^ (27) = 41.54, *p* < 0.001, RMSEA = 0.092, 90% CI [0.002–0.147], CFI = 0.958, SRMR = 0.086. All factor loadings (see Figure 1a) were statistically significant, with the exception of the indicator of pain. Given this result, a new CFA was estimated, dropping this indicator, and this new model (see Figure 1b) fit the data extremely well: χ^2^ (20) = 21.04, *p* < 0.001, RMSEA = 0.029, 90% CI [0.000–0.116], CFI = 0.997, SRMR = 0.063. In this model, all factor loadings were statistically significant and adequate.

Another CFA model was specified for the six charts of the COOP/WONCA. Again, a single latent variable of quality of life explained the six domains, and a graphical representation is shown in Figure 1c. This model fit the data extremely well, with all factor loadings being statistically significant and large: χ^2^ (9) = 8.69, *p* = 0.465, RMSEA = 0.000, 90% CI [0.000–0.141], CFI = 0.999, SRMR = 0.048.

### 3.3. Reliability

Coefficients alpha for internal consistency were 0.95 for the version of 9 domains of the COOP/WONCA and 0.93 for the 6 domains version. Means and standard deviations for each domain are shown in Table 3. Means ranged from a minimum of 1.10 (daily activities) to a maximum of 1.90 (general health status). Scores were calculated on a 1 to 5 scale, with higher values indicating worse quality of life.

### 3.4. Criterion-Related and Differential Validity

To gather validity estimates, each of the 9 domains of the COOP/WONCA was correlated with sociodemographic, clinical and physical variables. Additionally, the score of the COOP/WONCA was correlated with the SF-36 questionnaire, which is the specific measure of health used more frequently in patients with a stroke.

As for relationships among COOP/WONCA domains and sociodemographic and clinical variables, there were positive relations between age and physical function (r = 0.25; *p* < 0.05), daily activities (r = 0.21; *p* < 0.05), and general health status (r = 0.29, *p* < 0.01). Body Mass Index had a negative correlation with the feelings domain (r = −0.31, *p* < 0.01) but a positive one with changes in health status (r = 0.22; *p* < 0.05). Regarding MMSE, we found negative relations with physical condition (r = −0.35; *p* < 0.01), daily activities (r = −0.33; *p* < 0.01), social activities (r = −0.29; *p* < 0.01), health status (r = −0.26; *p* < 0.05), and social support (r = −0.29; *p* < 0.01), whereas the correlation was positive with the domain of change in health status (r = 0.49; *p* < 0.01). We also estimated mean differences as a function of gender, and we found significant differences in the domain of feelings (*p* < 0.01), daily activities (*p* < 0.05), and change in the health status (*p* < 0.05), with women scoring consistently lower than men.

There were no significant differences in the means of the COOP/WONCA domains as a function of type of stroke. However, time post-stroke had a negative correlation with the domains of physical condition (r = −0.34; *p* < 0.01), feelings (r = −0.29; *p* < 0.01), daily activities (r = −0.46; *p* < 0.01) and general health status (r = −0.34; *p* < 0.01). On the other hand, time post-stroke correlated positively with change in health status (r = 0.322; *p* < 0.01). When affected sides were compared in the means of the COOP/WONCA domains, we found statistically significant differences in social activities (*p* < 0.01), changes in health status (*p* = 0.003), pain (*p* < 0.01), and quality of life (*p* < 0.01). For all these domains, left-sided hemiparesis showed worse scores, except in the social activity domain, where worse scores were observed for the right side. Shoulder motor control had statistically significant mean differences for the domains of physical fitness (*p* < 0.01), social activities (*p* < 0.05) and change in health status (*p* < 0.05). Those subjects without motor control of their shoulders had a worse change in health status. Table 4 shows means, standard deviations and statistical significance for all the analyses.

We have also studied the relations of COOP/WONCA with physical tests. The Barthel Index was positively associated with physical condition (r = 0.63; *p* < 0.01), daily activities (r = 0.62; *p* < 0.01), and change in health status (r = 0.30; *p* < 0.01). Additionally, there were negative relations with social activity (r = −0.39; *p* < 0.01), general health status (r = −0.34; *p* < 0.01), social support (r = −0.26; *p* < 0.05) and quality of life (r = 0.24; *p* < 0.05). Hand function presented a negative association with physical condition (r = −0.26; *p* < 0.05), feelings (r = −0.27; *p* < 0.01) and social activity (r = −0.31; *p* < 0.01). Regarding wrist range of motion, the resting angle was positively associated to physical condition (r = 0.22; *p* < 0.05). Both active and passive wrist extension were negatively related to the domain of social support: r = −0.27 (*p* < 0.05) and r = −0.34 (*p* < 0.01), respectively. The resting angle of the metacarpophalangeal joints (MCP) of the fingers had a positive association with health status (r = 0.33; *p* < 0.01) and quality of life (r = 0.26; *p* < 0.05), and active extension of the MCP of the fingers had a positive association with daily activities (r = 0.24; *p* < 0.05) and negative with change in health status (r = −0.30; *p* < 0.01) and social support (r = −0.26; *p* < 0.05). Grip strength presented negative relationships with the domains of feelings (r = −0.28; *p* < 0.01), daily activities (r = −021; *p* < 0.05), and quality of life (r = −0.26; *p* < 0.01). Pinch strength had negative relationships with physical form (r = −0.22; *p* < 0.05), feelings (r = −0.40; *p* < 0.01), social support (r = −0.21; *p* < 0.05), and quality of life (r = −0.25; *p* < 0.01), but it had positive relations with the domain of change in health status (r = 0.26; *p* < 0.05). Regarding the associations with hypertonia, increased muscle tone in the elbow flexors presented a negative association with the general health status (r = 0.26; *p* < 0.05), and increased muscle tone in wrist flexors had a positive association with daily activities (r = 0.21; *p* < 0.05) and social activities (r = 0.31; *p* < 0.01), but a negative correlation with change in health status (r = −0.30; *p* < 0.01). Finally, increased muscle tone in the finger flexors was negatively associated with change in health status (r = −0.26; *p* < 0.05). Table 5 shows the correlations of the different sociodemographic, clinical and physical measures.

Finally, we calculated the association among the SF-36 questionnaire and domains of the COOP/WONCA, both of them measures of quality of life related to health. Physical condition in the SF-36 correlated significantly with the COOP/WONCA domains of physical condition (r = −0.502; *p* < 0.01), daily activities (r = −0.45, *p* < 0.01) and health status (r = −0.43; *p* < 0.01). Role—physical of the SF-36 also negatively correlated with daily activities (r = −0.42; *p* < 0.01) of the COOP/WONCA. Mental health dimension in the SF-36 negatively correlated with social activity (r = −0.40; *p* < 0.01), health status (r = −0.47; *p* < 0.01), and quality of life (r = −0.50; *p* < 0.01). The dimension of role—emotional in the SF-36, meanwhile, was significantly associated to feelings (r = −0.47; *p* < 0.01). With regard to the domain of social functioning of the SF-36, the significant associations with the dimensions in the COOP/WONCA were as follows: r = −0.65 (*p* < 0.01) with social activity; r = −0.54 (*p* < 0.01) with health status; and r = −0.49 (*p* < 0.01) with quality of life. Vitality in the SF-36 correlated negatively with physical condition (r = −49; *p* < 0.01), social activity (r = −0.47; *p* < 0.01), health status (r = −0.75; *p* < 0.01), and quality of life (r = −0.51; *p* < 0.01). General health in the SF-36 had negative relations with physical condition (r = −0.53; *p* < 0.01), social activity (r = −0.42; *p* < 0.01), health status (r = −0.80; *p* < 0.01), and quality of life (r = −0.62; *p* < 0.01) of the COOP/WONCA. Finally, bodily pain in the SF-36 also correlated negatively with several dimensions in the COOP/WONCA scale, specifically with health status (r = −0.41; *p* < 0.01), pain (r = −0.78; *p* < 0.01), and quality of life (r = −0.40; *p* < 0.01). All the aforementioned results are shown in Table 6.

## 4. Discussion

Quality of life is influenced by complex interactions between personal and environmental elements. Thus, the concept of quality of life results from a combination of objective and subjective aspects, involving factors related to economic and social development, standard of living, basic human needs, economic and social status, lifestyle, and health level, as well as patients’ perceptions of their overall living conditions and their degree of satisfaction with achievement. The assessment of quality of life allows not only the analysis of outcomes in clinical trials, disease monitoring, and evaluation of treatment response, but also the detection of the real needs of a population and, therefore, the appropriate planning of health intervention measures and resource allocation [49], along with personal aspirations [50].

There is a wide variety of tools aimed at assessing quality of life, but it is important to have instruments that are easy to administer and understand, reliable, and valid [11]. In this case, the COOP/WONCA charts were tested, showing good reliability and validity characteristics to be used as a HRQoL instrument in Spanish stroke survivors.

Considering the characteristics of the studied sample, the sample size was n = 91, compared to n = 55 in the only study of the COOP/WONCA charts conducted in an Irish stroke population [32]. Only patients under 18 years old and those with moderate-to-severe cognitive impairment were excluded due to the need for patient cooperation in responding to questionnaires and performing the battery of physical tests. Moreover, no time limits were set regarding the evolution of the stroke in order to obtain a more heterogeneous sample, including patients in different phases—acute, subacute, and chronic. Over half of the sample were in the chronic phase (more than 6 months post-stroke), while a small percentage (2.2%) were in the acute phase.

Our sample was similar to that used by Lennon et al. [32] in terms of mean age (68 years in our study versus 64 years in theirs), with 24% having aphasia and 24% presenting mild-to-moderate cognitive impairment. However, in our study, the majority were men (60.4%), in contrast to Lennon et al.’s (71% women, 57% men). Additionally, in our study, most participants had left-side impairment (58.2%), whereas Lennon et al.’s study had 57% with right-side impairment.

Regarding reliability, the COOP/WONCA charts proved to be a reliable measure for assessing quality of life in stroke patients, as demonstrated by other authors in the same subpopulation with a Cronbach’s alpha of 0.72 [32], in other populations with neurological conditions such as multiple sclerosis (α = 0.8) [31], and in chronic illness populations like hemodialysis patients (α = 0.76) [20] or those with chronic obstructive pulmonary disease (α = 0.89) [51].

In our study, reliability was calculated for both 9 and 6 domains, as the Spanish version uses 9 domains, while internationally, versions with 6 or 9 domains are used. Among the 19 validation studies reviewed, 10 used 6 domains, including stroke [32], multiple sclerosis [31], and cardiac transplant candidates [23]; 3 used 7 domains, including COPD [51] and Spanish osteoarthritis patients [19]; and 6 studies used 9 domains, 5 of which were Spanish, including a cross-cultural adaptation [18], a telephone-administered version [52], and two hemodialysis studies [20,53]. The only non-Spanish study with 9 domains was in a Persian multiple sclerosis population [30]. Therefore, we deemed it relevant to obtain results for both versions. We found that reliability was adequate in both cases, with Cronbach’s alpha values of 0.955 for 9 domains and 0.935 for 6 domains, both well above the threshold of 0.7. A measure is reliable insofar as it provides consistent results. In our case, we analyzed internal consistency via Cronbach’s alpha, which confirmed that the items are highly interrelated. However, temporal stability was not assessed, as in the measurement of Pedrero-Pérez et al. [52]. In terms of validity, analyses focused on differential and convergent construct validity. Sociodemographic variables showed that older age was associated with better scores in physical function, daily activities, and health status. Higher Body Mass Index was associated with worse scores in the feelings domain but better scores in general health. The strongest relationship was found with cognitive level: higher MMSE scores were linked to better quality of life. Women scored slightly lower than men, contrary to findings from other studies [52].

Stroke-related variables were significantly associated with at least one domain, except for stroke type (ischemic vs. hemorrhagic). Left-sided hemiparesis showed worse results in domains such as health status, pain, and quality of life, whereas right-sided hemiparesis was associated with worse results in social activities. This could be due to the typical motor-sensory and cognitive alterations caused by lesions in the affected cerebral hemisphere. Right hemisphere strokes can lead to left hemiplegia and spatial neglect, while left hemisphere strokes often cause right hemiplegia and aphasia [54]. As expected, patients without shoulder motor control had worse outcomes. Time post-stroke showed the strongest relationship, with longer duration linked to worse scores in physical fitness, feelings, daily activities, and general health.

In terms of associations between the proposed instrument and physical tests, the strongest correlations were found with the Barthel Index, significantly correlating with five domains (physical fitness, daily activities, change in health status, general health, and social activity) and weakly with two (social support and quality of life). Hand function showed a negative relationship with physical fitness, feelings, and social activity. Range of motion in the hand and fingers had a weak association with few domains. Grip and pinch strength were strongly related to feelings and quality of life. Grip strength also related to daily activities; pinch strength related to physical fitness and social support. Muscle tone had a low association in the elbow, wrist, and fingers, the strongest being wrist joint tone related to social activity and health status change.

Both the COOP/WONCA charts and the SF-36 are HRQoL questionnaires. The SF-36 is currently the most widely used stroke-specific questionnaire, supported by numerous publications confirming its psychometric quality [35]. The physical function domain of the SF-36 had a strong correlation with the physical fitness domain of the COOP/WONCA charts (r = −0.502; *p* < 0.01), consistent with other authors (r = −0.68; *p* < 0.01) [20]. The physical domain, assessing limitations in work or daily activities due to physical health, showed no strong correlation with any COOP/WONCA domain, again in line with these authors. The emotional role domain strongly correlated with the feelings domain, unlike some studies linking mental health with feelings [20,25,30]. In our study, mental health correlated with several domains such as social activity, health status, and quality of life. The bodily pain domain had its strongest correlation with the pain domain (r = −0.779; *p* < 0.01), aligning with other authors (r = −0.797; *p* < 0.01) [51]. Vitality and general health domains had their strongest correlation with the COOP/WONCA health status domain. Other authors found associations between general health and health status but not vitality [20,51]. Lastly, the SF-36’s social functioning domain had its strongest correlation with the COOP/WONCA social activity domain [20,30]. Although, a strong relationship was also found with general health, in line with one study [23].

Among the limitations of this study is that the reliability regarding temporal stability was not tested, as only one measurement was made at a single time point. Also, despite obtaining significant results, we believe a larger sample would have yielded results with greater statistical power. Future research could aim to analyze test–retest reliability across all dimensions at different time points, assess ceiling–floor effects across domains, and evaluate the scale’s sensitivity to change in intervention groups of post-stroke individuals.

## 5. Conclusions

The questionnaire appears to be a feasible tool for use with stroke populations, with high participant adherence. Compared to other scales, it provides a simpler, quicker, and easier application, as shown in previous studies. The COOP/WONCA charts are a useful HRQoL tool in stroke rehabilitation where time, communication, and concentration limitations may restrict the use of condition-specific scales.

In summary, the Spanish version of the COOP/WONCA charts is presented as a reliable and valid tool for use in individuals after stroke, with potential clinical utility as a measure of health-related quality of life. However, further studies are needed to assess additional psychometric properties of the scale.

## Figures and Tables

**Table 1:** Main characteristics of the participants: qualitative variables.

Variables		n (%)
Gender	Male	55 (60.4)
	Female	36 (39.6)
Ictus type	Hemorrhagic	27 (30)
	Ischemic	63 (70)
Hemiparesis	Right	38 (41.8)
	Left	53 (58.2)
Pain	Yes	52 (57.1)
	No	39 (42.9)
Aphasia	Yes	9 (9.9)
	No	82 (90.1)
Shoulder motor control	Yes	55 (60.4)
	No	36 (39.6)

**Table 2:** Main characteristics of the sample: quantitative variables.

Variables	Mean ± DT	Minimum–Maximum
Age	68.84 ± 9.44	44–93
BMI	26.16 ± 3.22	20.70–36.52
Time post-stroke (months)	23.60 ± 41.39	1–216
MMSE	28.64 ± 4.15	19–35
Barthel Index	66.04 ± 20.24	30–100
Box and Block Test	7.58 ± 8.84	0–31
Grip strength	6.57 ± 6.77	0–24

BMI: Body Mass Index; MMSE: Mini-Mental State Examination.

**Table 3:** Statistics of the COOP/WONCA dimension scores.

Variables	Mean ± DT
Physical form	1.68 ± 1.584
Feelings	1.26 ± 1.291
Daily activities	1.10 ± 1.266
Social activity	1.20 ± 1.457
Change in health	1.66 ± 1.423
General health	1.90 ± 1.799
Pain	1.22 ± 1.542
Social support	1.20 ± 1.212
Quality of life	1.44 ± 1.327

**Table 4:** Mean comparisons in the domains of the COOP/WONCA as a function of sample characteristics: *p*-values, means and standard deviations (between brackets).

		Physical Condition	Feelings	Daily Activities	Social Activity	Health Change	Health Status	Pain	Social Support	Quality of Life
Gender	*p*	0.192	0.005 **	0.044 *	0.503	0.019 *	0.146	0.694	0.206	0.132
	Male		2.44 (0.89)	2.53 (1.18)	2.22 (1.18)	2.27 (0.67)	3.20 (0.75)	2.15 (1.26)	1.75 (0.77)	2.35 (0.67)
	Female		3.06 (1.14)	3.00 (1.09)	2.36 (1.15)	2.64 (0.76)	3.44 (0.80)	2.25 (1.29)	1.97 (0.91)	2.50 (0.69)
Ictus type	*p*	0.211	0.431	0.615	0.815	0.308	0.339	0.205	0.876	0.333
	Hemorrhagic		2.56 (0.89)	2.67 (1.33)	2.22 (1.15)	2.52 (0.97)	3.41 (0.84)	1.96 (0.94)	1.81 (0.87)	2.52 (0.80)
	Ischaemic		2.75 (1.10)	2.76 (1.08)	2.29 (1.18)	2.37 (0.60)	3.29 (0.70)	2.29 (1.39)	1.83 (0.81)	2.37 (0.63)
Hemiparesis	*p*	0.286	0.558	0.174	0.004 **	0.003 **	0.376	0.004 **	0.181	0.009 **
	Right		2.61 (0.88)	2.89 (1.15)	2.68 (1.23)	2.21 (0.77)	3.21 (0.77)	1.76 (0.99)	1.97 (0.91)	2.21 (0.52)
	Left		2.74 (1.14)	2.58 (1.16)	1.98 (1.02)	2.57 (0.66)	3.36 (0.78)	2.49 (1.36)	1.74 (0.76)	2.55 (0.74)
Pain	*p*	0.533	0.260	0.607	0.098	0.126	0.673	0.000 **	0.150	0.304
	Yes		2.79 (0.99)	2.77 (1.07)	2.42 (1.14)	2.52 (0.77)	3.33 (0.73)	2.92 (1.04)	1.73 (0.81)	2.46 (0.72)
	No		2.54 (1.09)	2.64 (1.28)	2.08 (1.17)	2.28 (0.64)	3.26 (0.85)	1.21 (0.80)	1.97 (0.84)	2.33 (0.62)
Shoulder motor control	*p*	0.044 *	0.064	0.053	0.041 *	0.029 *	0.328	0.052	0.065	0.319
	Yes		2.53 (1.03)	2.53 (1.13)	2.11 (1.21)	2.55 (0.53)	3.27 (0.75)	2.38 (1.40)	1.71 (0.80)	2.36 (0.70)
	No		2.92 (1.02)	3.00 (1.17)	2.53 (1.05)	2.22 (0.92)	3.33 (0.82)	1.89 (0.97)	2.03 (0.84)	2.47 (0.65)

Notes: * = *p* < 0.05; ** = *p* < 0.01.

**Table 5:** Correlations among the sociodemographic, clinical and physical variables with the domains of the COOP/WONCA.

	Physical Condition	Feelings	Daily Activities	Social Activity	Health Change	Health Status	Pain	Social Support	QoL
Age	0.254 *	0.113	0.214 *	0.148	−0.162	0.293 **	0.137	0.173	0.126
BMI	0.062	−0.314 **	−0.139	−0.100	0.222 *	−0.012	−0.122	−0.004	0.063
Time post-stroke (months)	−0.340 **	−0.296 **	−0.457 **	−0.188	0.322 **	−0.342 **	−0.202	0.087	0.001
MMSE	−0.351 **	−0.178	−0.333 **	−0.288 **	0.490 **	−0.266 *	0.063	−0.290 **	−0.073
Barthel Index	0.629 **	−0.174	0.627 **	−0.389 **	0.300 **	−0.339 **	0.073	−0.261 *	−0.240 *
Box and Block Test	−0.261 *	−0.276 **	−0.110	−0.069	0.149	0.095	0.163	−0.312 **	−0.165
Wrist resting angle	0.218 *	0.165	0.190	−0.163	0.097	0.201	0.172	−0.156	0.023
Wrist active extension	−0.088	−0.173	0.091	0.053	0.133	0.192	0.087	−0.268 *	−0.123
Wrist passive extension	−0.077	−0.178	0.130	0.096	0.058	0.121	−0.172	−0.338 **	−0.084
MCP resting angle	0.109	0.083	0.182	0.195	0.077	0.336 **	0.154	−0.128	0.262 *
MCP active extension	−0.070	−0.151	0.243 *	0.033	−0.299 **	−0.139	−0.137	−0.258 *	−0.109
Grip strength	−0.158	−0.284 **	−0.213 *	0.075	0.140	0.207	−0.198	−0.132	−0.260 *
Pinch strength	−0.215 *	−0.401 **	−0.160	0.078	0.264 *	0.017	−0.054	−0.213 *	−0.250 *
MAS score for elbow flexors	0.015	0.164	−0.035	0.120	−0.172	−0.210 *	0.022	0.113	0.031
MAS score for wrist flexors	0.105	0.122	0.207 *	0.311 **	−0.302 **	−0.100	0.002	0.065	−0.042
MAS score for MCP flexors	−0.005	0.148	−0.057	0.053	−0.262 *	−0.166	0.078	0.017	−0.017
MAS score for IP flexors	−0.049	0.207 *	−0.066	0.063	−0.175	−0.146	0.141	0.067	0.056

Notes: * = *p* < 0.05; ** = *p* < 0.01; BMI: Body Mass Index; MMSE: Mini-Mental State Examination; MAS: Modified Ashworth Scale; MCP: Metacarpophalangeal; IP: Interphalangeal.

**Table 6:** Correlations among the domains of the SF-36 and the dimensions of the COOP/WONCA.

	Physical Condition	Feelings	Daily Activities	Social Activity	Health Change	Health Status	Pain	Social Support	QoL
SF Physical functioning	−0.502 **	0.134	−0.449 *	−0.256	0.010	−0.430 *	−0.200	−0.195	−0.349
SF Role—physical	−0.259	−0.154	−0.415 *	−0.312	−0.240	−0.173	−0.059	−0.170	−0.346
SF Mental health	−0.056	−0.220	0.032	−0.402 *	−0.057	−0.472 **	−0.240	−0.110	−0.505 **
SF Role—emotional	−0.188	−0.472 **	−0.161	−0.130	−0.347	0.005	0.030	−0.280	−0.279
SF Social functioning	−0.287	−0.042	−0.122	−0.656 **	−0.159	−0.540 **	−0.243	−0.213	−0.495 **
SF Vitality	−0.490 **	0.149	−0.206	−0.472 **	−0.041	−0.753 **	−0.254	−0.219	−0.514 **
SF General health	−0.530 **	0.094	0.102	−0.422 *	−0.048	−0.804 **	−0.334	−0.117	−0.620 **
SF Bodily pain	−0.090	−0.018	−0.167	−0.361	−0.121	−0.406 *	−0.779 **	−0.215	−0.403 *

Notes: * = *p* < 0.05; ** = *p* < 0.01.

## Data Availability

The original contributions presented in this study are included in the article. Further inquiries can be directed to the corresponding author.
